# Health Monitoring of Civil Infrastructure and Materials

**DOI:** 10.1155/2014/435238

**Published:** 2014-02-18

**Authors:** Dimitrios G. Aggelis, Ninel Alver, Hwa Kian Chai

**Affiliations:** ^1^Department of Mechanics of Materials and Constructions, Vrije Universiteit Brussel, Pleinlaan 2, 1050 Brussels, Belgium; ^2^Department of Civil Engineering, Ege University, Bornova, 35100 Izmir, Turkey; ^3^Department of Civil Engineering, University of Malaya, 50603 Kuala Lumpur, Malaysia

Despite the generally long life span of concrete structures, they cannot be considered maintenance-free. Several incidents of catastrophic failures remind the engineering world that monitoring of structures is imperative nowadays both for prevention of loss of life and property and also for effective infrastructure management based on a usually finite budget. Due to the variety of structural sizes, shapes, and uses, as well the possible vulnerabilities of the different types of structures, different tools must be used, many times complementary, in order to lead to reliable assessment results. Indeed, the reliability of in situ implementation is a key issue for any health monitoring technique along with other characteristics such as testing speed and cost-effectiveness.

Several nondestructive testing (NDT) techniques have already been established, showing their suitability in certain aspects of material and structural characterization and behavior monitoring. As an example, the use of ultrasonic pulse velocity can be mentioned, the correlation of which with the quality of concrete is well documented. Other techniques based on elastic waves such as impact and impulse response, as well as the radar method, have proven their suitability in distinguishing delaminations and inhomogeneities. The assessment based on the above-mentioned methods as well as a number of others, like acoustic emission, radiography, vibration modal analysis, and slightly destructive surface strength methods in conjunction with visual inspection, provides a valuable platform for decision making concerning the maintenance, based on more robust engineering criteria than solely the experience of the engineer.

However, it is a common impression that most NDT techniques have not at all reached their full potential especially with regard to in situ implementation. Therefore, the current special issue intends to examine all possible tools for economic and timely infrastructure condition assessment, with emphasis on reliability and connection of the monitoring results with the proper maintenance action that should be taken. The challenges are even higher since new and innovative materials are being increasingly used. These materials include high performance concrete, textile reinforced concrete, and nanomodified and recycled materials, which offer better capabilities for sustainable structures; however their assessment through the same techniques used for conventional concrete should not be taken for granted.

In this special issue, the latest advances in different topics of civil structural health monitoring (SHM) are highlighted. More specifically, recent findings in elastic wave methods (acoustic emission, impact-echo, and ultrasonics) are reported. Vibration methodologies which are the most suitable for global bridge SHM are discussed, while the use of radar is reviewed and applied. Advancements in automated robotic inspection and online monitoring of bridge components are also discussed. The issue includes studies on corrosion detection, reinforcing and self-sensing elements for SHM. Algorithms incorporating pattern recognition clustering for the characterization of the location and degree of damage could not but be a strong part of the issue, while monitoring of self-healing and textile-reinforced materials is also discussed. The dual importance of monitoring of old structures is stressed out: their structural safety as well as their cultural heritage significance. The methodologies are successfully tested in several case studies as reported herein.

We believe that the present special issue reflects on the recent advances in SHM and NDT for civil structures and materials complemented with insightful findings of various assessment techniques. We wish to thank all the authors for submitting their work in the issue and their patience during the review process.

*Dimitrios G. Aggelis* *Dimitrios G. Aggelis* *Ninel Alver* *Ninel Alver* *Hwa Kian Chai* *Hwa Kian Chai*

